# The effect of autism phenotype and diagnosis disclosure on students’ desire for social distance from autistic peers

**DOI:** 10.1177/13623613241230128

**Published:** 2024-02-27

**Authors:** Indrė Muraškaitė, Kristina Žardeckaitė-Matulaitienė

**Affiliations:** Vytautas Magnus University, Lithuania

**Keywords:** autism, autism knowledge, contact, female phenotype, social distance

## Abstract

Autistic students report feeling lonely, rejected by peers which is associated with poorer well-being and lower academic results. This study aims to evaluate the effect of autism phenotype and diagnosis disclosure on students’ desire for social distance from autistic students and factors related to social distancing. 303 students (72.3% women; mean age = 22.37 years) participated in this study. Each participant was assigned to one of the six vignettes depicting students of different gender and diagnostic status. Respondents’ willingness to engage in different social situations, type and quality of prior contact with autism, autism knowledge and social desirability bias were measured. Results showed that students desired greater social distance from autistic males compared to autistic females, as well as from peers with non-disclosed diagnosis compared to those with disclosed diagnosis. Greater autism knowledge was related to less desire for social distance in non-disclosure, disclosure and in male autism phenotype conditions. Higher quality of contact was associated with less desire for social distance from autistic students with disclosed diagnosis. Type of contact was not related to social distance. Findings suggest that autistic male students and individuals with non-disclosed diagnosis might be more vulnerable to social exclusion.

**Lay abstract:**

Autistic students experience loneliness, rejection from peers, which might negatively affect both their well-being, as well as academic results. Others have studied this topic, however, the existing research does not analyze the desire for social distance from autistic female students in higher education. The goal of this study was to evaluate whether the way autism is expressed and disclosing the autism diagnosis had an effect on students’ willingness to interact with autistic male and female students. We also analyzed participants’ knowledge about autism, contact with autistic people in the past and its pleasantness in relation to their willingness to interact with autistic students described in the scenarios of the present study. We found that students were less willing to interact with autistic male students compared to autistic female students, as well as with autistic students whose diagnosis was not disclosed compared to autistic students whose diagnosis was disclosed to the raters. In addition, students who reported more pleasant contact with autistic individuals in the past were more willing to socially interact with autistic students if their diagnosis was disclosed. Students with higher knowledge of autism expressed greater willingness to interact with autistic males and autistic students regardless of diagnosis disclosure. Findings suggest that autistic males and individuals who prefer not to disclose their diagnosis are more vulnerable to social exclusion. More subtle expressions of autism need to be researched further.

## Introduction

Social distance is a behavioral component of stigma that describes the degree of willingness or avoidance to interact with an individual (Link et al., 2004). Within the framework of Social identity theory, autistic individuals may face stigmatization as they are seen as different from the majority group and considered “out-group” members (Stets & Burke, 2000). One of the key differences in autism manifests in the social area, which may lead to non-autistic students being less willing to interact with their autistic peers compared to others who display normative social behavior (Stockwell et al., 2021). In addition, non-autistic students rate autistic peers less favorably and perceive most of their characteristics as negative, potentially increasing the desire to maintain social distance (Sasson & Morrison, 2019; Underhill et al., 2019; Wood & Freeth, 2016). These experiences of social rejection, stigma and the sense of being different might lead autistic people to conceal non-normative behaviors, which can negatively impact their psychological well-being and psychosocial functioning (Botha & Frost, 2020; Cage et al., 2018; Perry et al., 2022). Hence, exploring various factors related to social distancing may be important for supporting the well-being of autistic students.

Research studies analyze different factors that could contribute to reducing stigmatization of autistic individuals. However, despite the heterogeneity of autism presentations, they have limited generalizability, as they predominantly represent males, with only a few attempts to explore different types of autism using gender-neutral vignettes (e.g. disruptive or withdrawn autism) (Gillespie-Lynch et al., 2021; Stockwell et al., 2021). Most researchers agree that autism might manifest differently in males and females, with autistic women masking their traits more compared to autistic men (Beggiato et al., 2017; Cook et al., 2021; Schuck et al., 2019). Autistic masking involves conscious or unconscious suppression of autistic traits and the implementation of different strategies to fit in, which stems from deficit narratives or stigmatization of autism (Pearson & Rose, 2021). Research on children and adolescents indicate that autistic girls are often viewed as more typical and receive more positive ratings (Cola et al., 2020; Geelhand et al., 2019). This suggests that social distancing behavior toward autistic female students may be different compared to autistic males, potentially leading to distinct support needs. However, it remains unknown how autistic females are perceived in higher education and whether non-autistic students are more willing to interact with them. Although we do not support the idea that there is only one type of female autism, in this study, we will explore one possible set of characteristics typically associated with the autistic female phenotype.

Autistic students’ needs may also vary regarding their willingness to disclose their autistic identity. Anderson et al. (2017) showed that the most common reason of not disclosing the autism diagnosis in post-secondary education settings was a fear of being stigmatized. Attribution theory, on the contrary, suggests that disclosure should improve acceptance by attributing non-normative behaviors to uncontrollable factors (e.g. neurological) rather than assigning the responsibility for a condition to a person (Weiner, 1993). Research confirms that non-autistic peers’ responses to autism disclosure are usually positive and may reduce social distancing, however, for many higher education students hiding an autism diagnosis is another way to avoid stigma and “*pass as normal”* (Anderson et al., 2017; Brosnan & Millis, 2016; Matthews et al., 2015). Given that some are hesitant to disclose, it is crucial to examine stigma experienced by autistic students who conceal their diagnosis, especially across different genders.

## The present study

To fill the existing research gap and meet the diverse needs of autistic students, we added two categories to the analysis of social distance and its related factors. First is the female autism phenotype which represents a less typical expression of autism and includes some characteristics of autistic masking. Second is the diagnosis disclosure as hiding the diagnosis may be another way to avoid potential stigma (Anderson et al., 2017). Our primary aim was to assess whether the autism phenotype and the disclosure of an autism diagnosis affected students’ desire to socially distance from autistic peers presented in hypothetical scenarios. As prior studies on the relationships between autism knowledge, contact type and quality, and intentions to socially distance from autistic students have shown inconsistent results and have not been well-studied in relation to various presentations of autism (Gillespie-Lynch et al., 2015, 2021; Kitchin & Karlin, 2022; Link & Phelan, 2001; Lu et al., 2022; Matthews et al., 2015; Obeid et al., 2015; Someki et al., 2018; White et al., 2019), we sought to additionally investigate the potential associations between these factors and social distancing across different phenotype and disclosure conditions. We anticipated that students would desire more social distance from autistic males and autistic students with disclosed diagnosis, as opposed to autistic females and autistic students with non-disclosed diagnosis. Furthermore, we expected autism knowledge, having prior contact with autism and the quality of that contact to be related to the reduced desire for social distance from autistic students.

## Methods design

Quantitative quasi-experiment method based on vignettes was selected for the research. A 2 × 2 between-subjects design where each participant is assigned to only one vignette was used. There were two independent variables: (1) type of autism presentation (male phenotype vs female phenotype) and (2) diagnosis disclosure (diagnosis disclosed vs diagnosis not disclosed). The study consisted of two parts. In the first part, the effect of independent variables on the dependent variable (social distance) was measured. In the second part, other factors’ (autism knowledge, type, and quality of prior contact with autism) relationship with the social distance scores were assessed.

## Participants

Participation occurred in two stages. A total of 303 students (219 women, 83 men, one other) from different Lithuanian universities and colleges took part in the first part of the survey. 19 participants refused to continue into the second part or were excluded due to incorrect or missing data. The final sample of the second part of the study consisted of 284 students (198 women, 71 men, one other). The age of participants in the study varied between 18 and 45 years (*M* = 22.38, *SE* = 4.1). See Table 1 for sample characteristics in Part 1 and Part 2 of the study.

**Table 1.** Sample characteristics.

	Part 1 (*N* = 303) *N* (%)	Part 2 (*N* = 284) *N* (%)
Gender^a^		
Female	219 (72.3)	208 (73.2)
Male	83 (27.4)	75 (26.4)
Other	1 (0.3)	1 (0.4)
Higher education institution type		
University	263 (86.8)	248 (87.3)
College	40 (13.2)	36 (12.7)
Year of study		
First to Second year (Bachelor’s degree)	139 (45.9)	132 (46.5)
Third to Fourth year (Bachelor’s degree)	117 (38.6)	109 (38.4)
First to Second year (Master’s degree)	42 (13.9)	39 (13.7)
Doctoral studies	5 (1.7)	4 (1.4)
Academic major		
STEM	71 (23.4)	64 (22.5)
Helping professions	93 (30.7)	90 (31.7)
Other	139 (45.9)	130 (45.8)
Prior contact		
Yes	94 (31.0)	94 (32.1)
No	193 (63.7)	190 (66.9)

STEM: science, technology, engineering, mathematics.

a There were significant differences in gender across six study conditions (*p* < .05).

Participants were recruited by sending invitations to student representatives and scientific associations of different higher education institutions in Lithuania, as well as by posting advertisements on social media and student *Facebook* groups. Eligible participants had to be aged over 18 and enrolled at a Lithuanian university or college. Participation was voluntary, not rewarded. Written informed consent was obtained from all participants. At the time of the study, *the Psychologists’ Professional Ethics Board at Vytautas Magnus University* only mandated formal ethical approval for research undertaken by faculty researchers, with no such requirement for student-led studies. Given that the main author of the study was a student, and the research was conducted within a general population and presented no or minimal risk to participants, the Ethics Committee at Vytautas Magnus University concluded that formal ethical approval was not a requisite for this particular study. To ensure compliance with the ethical standards outlined in the Declaration of Helsinki and in accordance with the general recommendations established by *the Psychologists’ Professional Ethics Board at Vytautas Magnus University*, a co-author of the research, who is a committee member of the university- based Ethics Committee, guided every stage of planning and implementing the study.

## Materials

### Vignettes

#### Vignettes development

Taking the novelty of the study into account, a total of six vignettes were developed for the study. Four experimental groups: (1) autistic male student (disclosed diagnosis); (2) autistic female student (disclosed diagnosis); (3) autistic male student (diagnosis not disclosed); (4) autistic female student (diagnosis not disclosed), two control conditions (5) neurotypical male; (6) neurotypical female were created. Methodological guidelines made by Evans and colleagues (2015) were followed to ensure the validity of the stimulus material. All vignettes were presented in a written format (176-262 words each) and depicted a typical student dormitory environment. Descriptions of autistic students were based on Matthews et al. (2015) vignettes No. 1 and No. 2. The ICD-10 diagnostic criteria for Asperger’s syndrome were used to illustrate the social interaction features (e.g. difficulties in making and maintaining friendships or understanding social cues) and intense, focused interests (e.g. expertise in the field of interest, tendency to collect, categorize objects). Traits representing (non)verbal communication were based on Adult Asperger Assessment criteria (AAA, Baron-Cohen et al., 2005), this includes the tendency to redirect a conversation toward one‘s own topic of interest and a highly detailed manner of speaking. Traits and behaviors typical to *autistic male phenotype* (i.e. more typical representation of autism) or *autistic female phenotype* (i.e. less typical type of autism) were drawn from gender-related autism research. For instance, autistic males were portrayed as having interest in technology, video games, while autistic females were depicted as passionate about literature. In addition, autistic female students were presented as quiet, withdrawn, struggling with initiating contact, yet more socially active than autistic males. The vignettes also featured academically capable students (e.g. assisting others with university assignments) who were verbal and exhibited sensory differences. The disclosure statement was added to the conditions where diagnosis was disclosed: “Autism and its spectrum disorders (ASD) are a group of complex developmental disorders characterized by distinctive social interaction, communication and behavioral characteristics.” In non-disclosure conditions, participants were not provided the information on whether the student in the description was neurotypical or autistic. The neurotypical students’ conditions served as comparison groups to ensure that any changes in dependent variable were caused by independent variables and not by other unrelated factors. These vignettes represented introverted, rather shy, yet typically developing male or female students. All vignettes were in Lithuanian and are available under request.

#### Vignettes validity

To enhance the validity of vignettes, a panel of five independent experts (one psychiatrist, three psychologists, and one autistic mother of an autistic child) was formed. Everyone had professional or lived experience with autistic adults of both genders. They were asked to rate five randomly set and non-labeled vignettes on a seven-point Likert-type scale using the adapted McCrow et al. (2013) questionnaire based on five factors: (1) plausibility; (2) clarity; (3) simplicity; (4) construct validity; (5) diagnostic agreement. If needed, raters could leave additional comments in each category. The overall inter-rater score after the first review reached the recommended minimum of .90 (S-CVI/Ave = .90) (Waltz et al., 2005). Nevertheless, following the thematic analysis of experts’ comments, further adjustments were made in order to create more realistic and less stigmatizing descriptions. Three out of five raters took part in the second evaluation stage, which is the minimal acceptable number to compute the S-CVI score (Lynn, 1986). Raters were given the same questionnaire, yet the order of the revised vignettes was changed. Overall agreement among experts after the re-review was high (S-CVI/Ave = .995)—three vignettes scored S-CVI/Ave of 1, only the autistic female vignette had a somewhat lower rating of .98.

## Measures

### Demographic questions

Participants were asked to report their age, gender, academic major, year of study, and name of the higher education institution.

### Social distance

Social distance was measured using the adapted Bogardus (1933) Social distance scale (Gillespie-Lynch et al., 2021). The scale consists of 10 statements that assess the research participants’ willingness to engage in different social situations with the student described in the vignette. Social situations differ from each other by the degree of closeness. Participants are asked to rate each statement on a five-point Likert-type scale from −2 (*strongly agree*) to +2 (*strongly disagree*). The total score could range from −20 to +20 with higher scores representing a greater desired social distance. Originally, the scale was used to study stigma in employment, therefore the statements in the questionnaire related to the work environment were adjusted to match the context of the current research (e.g. the statement “I would NOT be willing to have an autistic co-worker” was changed to “I would NOT be willing to have X as my course mate”). In this study, the internal consistency of the scale was strong (α = .83).

### Contact type

Participants’ past experience of prior contact with autistic individuals was assessed with one item adapted from Gillespie-Lynch et al. (2021) study: “Please select as many of the following types of relationships as you’ve had with autistic people (e.g. your child, your friend, your spouse).” Given that some respondents may not have had prior contact with autistic individuals, the additional option “I have no experience of personal contact with autistic individuals” was added. Participants who had any kind of personal experience with autistic individuals were classified as having prior contact with autism.

### Contact quality

Participants were asked to rate the pleasantness of the prior contact with autistic individuals using an item adapted from Gardiner and Iarocci (2014): “In the past, your experiences with autistic individuals have been pleasant” (Gillespie-Lynch et al., 2021). Responses were scored on a seven-point Likert-type scale ranging from 1 (*strongly disagree*) to 7 (*strongly agree*). A higher score indicated a more pleasant experience.

### Knowledge of autism

Participatory Autism Knowledge Scale (Gillespie-Lynch et al., 2021) adapted from the Autism Knowledge Questionnaire (AKQ; Stone, 1987) was used to measure participants’ knowledge about autism. This tool was employed in the current research study since it reflected the newest changes in diagnostic criteria and included items regarding autism in adulthood and autistic masking. The scale consisted of 29 statements that participants were asked to rate on a five-point Likert-type scale ranging from −2 (*strongly disagree*) to +2 (*strongly agree*). The overall score varied from −58 to +58 with a higher score representing better knowledge about autism. Internal consistency in this sample was robust (α = .82). The permission to use the instrument was obtained via correspondence with the first author.

### Social desirability

To avoid socially desirable answers, the Balanced Inventory of Desirable Responding (BIDR) (Paulhus, 1991) was used as it showed good psychometric characteristics in Lithuania (Genevičiūtė-Janonienė, 2015). The measure consisted of 20 statements that fell into two subscales—self-deception (10 statements) and impression management (10 statements). Participants rated each item on a seven-point Likert-type scale ranging from 1 (*strongly disagree*) to 7 (*strongly agree*). Higher scores indicated a greater tendency to provide socially desirable responses. In this study, the overall internal consistency of the scale was satisfactory (α = .69).

## Procedure

The survey was conducted online using *Google Forms*. Following the informed consent form, participants filled out a demographic questionnaire and were asked to choose a number from 1 to 6. Based on the selected number, each participant was assigned to one of the 6 vignettes depicting students of different gender (male or female) and diagnostic status (neurotypical/autism disclosed/autism non-disclosed).

In the first part of the study, respondents rated their desire for social distance from a student depicted in a vignette in situations of varying degrees of closeness and filled out the social desirability questionnaire. At the end of the first part, participants read a debriefing statement, which included a more specific goal of the study and disclosed whether the student depicted in the vignette was neurotypical or autistic. Participants assigned to neurotypical or autistic students with a non-disclosed diagnosis conditions were presented with the definition of autism identical to that given in disclosure conditions. To ensure the validity of the results, respondents were asked not to share the information regarding the survey with other eligible participants who might take part in the study. Participants who agreed to progress to the second part of the survey were asked to report all the types of relationships they have had with autistic people in the past and rated its quality. Finally, participants filled out the autism knowledge questionnaire. Research data were collected in March and April 2021.

## Analytic approach

The data analysis was performed using IBM SPSS Version 26. The scores on the Social distance scale, Participatory Autism Knowledge questionnaire and the Balanced Inventory of Desirable Responding measure were normally distributed, therefore parametric statistics were used for the analysis. The Chi-square test (for categorical and ordinal variables) and analysis of variance (ANOVA) (for continuous variables) were used to assess the distribution of baseline characteristics across different experimental groups. Due to uneven distribution of gender variable, both gender and social desirability variables were controlled in conducting statistical analysis of collected data (see Table 1). One-way analysis of covariance (ANCOVA) was used to compare mean differences. Partial correlation analysis was used to determine the relationship between social distance and knowledge of autism or quality of contact variables. The significance level selected for the study was .05.

## Community involvement

The first author, who is autistic, created the preliminary methodology for the study. Members of the Lithuanian online community for autistic adults contributed by providing comments on the descriptions of autistic adult students depicted in the vignettes. Five professionals in the field, including an autistic mother of an autistic child, helped to further develop the study materials. The expert panel reviewed both the primary and the final versions of the vignettes and provided suggestions for adjustments.

## Results

### Preliminary analyses

The initial analysis showed that there were no significant differences in distribution of baseline characteristics across six groups, except the social distance variable (see Table 2). The overall social distance score could range from −20 to +20. Participants expressed the greatest desire for social distance in an autistic male with non-disclosed diagnosis condition. The least desire for social distance was observed in a neurotypical female condition.

**Table 2.** Baseline characteristics.

	Full sample Mean (*SD*)	1	2	3	4	5	6
	*N* = 303	*N* = 63	*N* = 59	*N* = 82	*N* = 38	*N* = 34	*N* = 27
Social distance	−7.6	−6.25	−3.83	−9.17	−9.37	−8.67	−10.41
	(5.88)	(6.32)	(4.99)^a^	(5.21)	(5.46)	(4.99)	(5.74)
Social desirability	81.66	81.16	79.73	80.76	85.97	85.21	79.26
	(15.19)	(14.93)	(15.82)	(15.1)	(14.54)	(15.8)	(14.94)
Autism knowledge	*N* = 284	*N* = 58	*N* = 55	*N* = 76	*N* = 36	*N* = 32	*N* = 27
	20.42	21.95	19.29	22.37	21.36	14.56	19.59
	(11.69)	(11.73)	(12.25)	(10.63)	(10.34)	(12.35)	(12.63)
Contact quality	*N* = 91	*N* = 19	*N* = 15	*N* = 26	*N* = 14	*N* *=* 8	*N* *=* 9
	4.99	5.11	5.13	5.19	4.79	4.63	4.56
	(1.46)	(1.33)	(1.80)	(1.27)	(0.89)	(2.01)	|(1.94)

1—Autistic male (diagnosis disclosed); 2—Autistic male (diagnosis non-disclosed); 3—Autistic female (diagnosis disclosed); 4—Autistic female (diagnosis non-disclosed); 5—Neurotypical male; 6—Neurotypical female.

a Significantly different from third, fourth, sixth conditions (*p* < .001), and fifth condition (*p* < .05).

### Autism phenotype and diagnosis disclosure effect on social distancing

A one-way ANCOVA was conducted to compare the effects of autism phenotype and diagnosis disclosure on social distancing scores. There was a significant effect of both phenotype, *F*(2, 296) = 13.620, *p* < .0001, and diagnosis disclosure, *F*(2, 297) = 7.09, *p* < .05, after controlling for gender and social desirability scores. Phenotype had between medium and large effect size (η^2^ = .12) while diagnosis disclosure had between small and medium effect size (η^2^ = .05).

#### Phenotype

**Figure 1.**
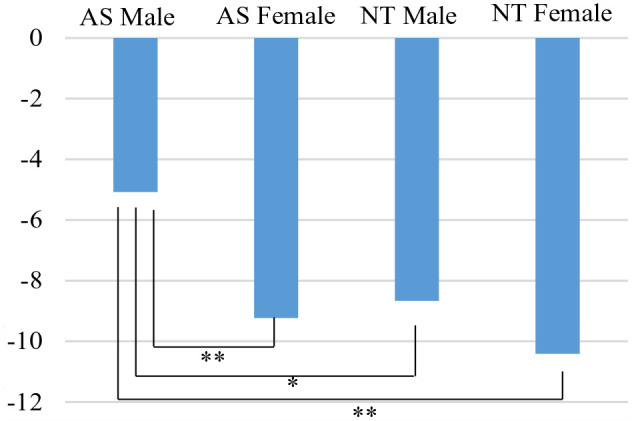
Social distance scores across phenotype conditions. AS = autistic; NT = neurotypical. *p < .001; **p < .05.

As shown in Figure 1, post hoc analysis using the Bonferroni test indicated that students reported greater social distance from autistic male students (*M* = −5.08, *SD* = 5.82) compared to autistic female (*M* = −9.23, *SD* = 5.27, *p* < .001), neurotypical male (*M* = −8.67, *SD* = 4.99, *p* = .007) and neurotypical female students (*M* = −10.41, *SD* = 5.74, *p* < .001). No significant differences were observed between autistic female and neurotypical male or female conditions (both *p*s > .05).

#### Diagnosis disclosure

**Figure 2.**
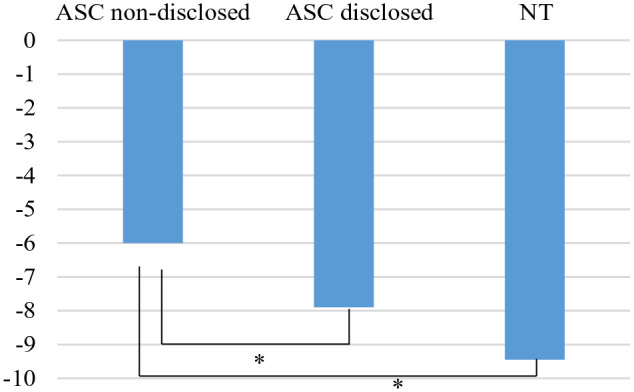
Social distance scores across diagnosis disclosure conditions. ASC = autism spectrum condition; NT = neurotypical. *p < .001; **p < .05.

Students expressed a greater desire for social distance from autistic students with non-disclosed diagnosis (*M* = −6, *SD* = 5.82) compared to autistic students with diagnosis disclosed (*M* = −7.90, *SD* = 5.88, *p* = .04) and neurotypical students (*M* = −9.45, *SD* = 5.36, *p* = .001). The difference between students’ desire to socially distance themselves from neurotypical students and autistic students with diagnosis disclosed was not determined (*p* = .21) (see Figure 2).

### Autism knowledge, contact type, and quality relationship with social distancing

#### Knowledge about autism

Controlling for gender and social desirability scores, a negative correlation between autism knowledge and social distancing was found in both autism disclosure and non-disclosure conditions, and in autistic male phenotype condition (see Table 3).

**Table 3.** Bivariate correlations across different study conditions.

Condition	AS Male	AS Female	NT Male	NT Female	AS disclosed	AS non-disclosed	NT
Social distance							
Autism knowledge	−.60**	−.18	.06	.56	−.37*	−.47*	−.23
Contact quality	−.17	−.31	.37	.57	−.39*	−.04	−.30

AS = autistic; NT = neurotypical.

* *p* < .05; **p < .001.

#### Contact type and quality

As Table 3 shows, participants’ quality of prior contact with autistic individuals was negatively associated with desired social distance from autistic students with disclosed diagnosis. No other significant associations of prior contact with autism and social distancing were found. There were no significant differences in any of the conditions comparing social distancing scores between participants with or without prior contact with autistic individuals too.

## Discussion

This study contributes to the limited body of literature that addresses various types of autism presentations and, to our knowledge, is the first to examine social distancing from autistic female students in higher education. This study found that students expressed less social distance in autistic female student and diagnosis disclosure conditions compared to autistic male and non-disclosed diagnosis conditions. Interestingly, no differences were observed when analyzing social distancing from autistic females and neurotypical students of both genders, as well as when comparing the autism disclosure condition to neurotypical students’ condition. In addition, the autism phenotype had a close to large effect on participants’ desire for social distance from autistic students, which was greater than the effect size of diagnosis disclosure. Furthermore, contact quality but not its type was related to reduced social distance. Nevertheless, this relationship was significant only in disclosed autism condition. Finally, autism knowledge correlated with less social distancing in all conditions, except the autistic female condition. This suggests that previous research on the stigmatization of autistic male students or gender-neutral vignette characters cannot be readily generalized to autistic females, highlighting the need for more diverse social support programs.

It is important to note that the overall desire for social distance from autistic students was not high, ranging between the response categories “neither agree nor disagree” and “somewhat agree.” This is consistent with previous research that suggests less disruptive autistic traits may be less stigmatized compared to the phenotype with more prominent behavioral issues or other disorders, such as schizophrenia or psychopathy (Gillespie-Lynch et al., 2021; Cola et al., 2020; Obeid et al., 2021; Stockwell et al., 2021). Drawing from Jones (1984) six-dimensional model of stigma, the invisibility of autism, its neurobiological and genetic basis, and the fact that autistic traits in adults are usually less pronounced and less often associated with danger may reduce the stigma (Gillespie-Lynch et al., 2021; Tillmann et al., 2018). Thus, our findings may have been affected by socially acceptable characteristics of autism represented in the study. Such results imply that providing information about the etiology of autism and emphasizing positive characteristics could potentially contribute to reducing social distance.

This study extends upon research focused predominantly on male autism, and adds to other studies that explore various types of autism (Gillespie-Lynch et al., 2021; Matthews et al., 2015; Stockwell et al., 2021) by showing that students’ desire for social distance varies depending on how autism is expressed. The findings that students desired greater social distance from autistic males compared to autistic females, while there were no differences in social distancing between autistic females and neurotypical students may be explained by earlier studies showing that autistic women are viewed more positively due to their more normative behavior (Cola et al., 2020; Little et al., 2017; Whitlock et al., 2020). It also supports the Social Identity Theory, stating that appearing more similar to the “in-group” may reduce symbolic threat through social comparison and increase the willingness of *“out- group”* members to interact with a minority group (Stets & Burke, 2000). The behaviors represented by the male autism phenotype deviate significantly more from accepted norms, potentially placing them in the “out-group” category, thereby increasing the desire for social distance. Although students with less pronounced autistic traits may experience greater social acceptance, it is important to note that in some cases, it may result from efforts to conceal undesirable traits due to fear of stigma (Pearson & Rose, 2021; Perry et al., 2022). Thus, the results of autistic female students being rated similarly to non-autistic peers should be interpreted with caution, as concealing the “autistic-ness” may pose unique challenges.

As expected, students wanted more social distance from autistic peers who did not disclose their diagnosis compared to students who did disclose. These results align with research indicating people’s positive attitudes toward autism disclosure, despite the fear of stigma reported by autistic students (Anderson et al., 2017). Our study cannot identify the underlying mechanism behind this result; however, the Attribution theory suggests that the disclosure might activate certain attitudes (e.g. personal responsibility and perceived dangerousness), which affect people’s behavior (Link & Phelan, 2001; Weiner, 1993). Autism, especially the withdrawn type, is more often associated with intelligence rather than perceived dangerousness (Gillespie-Lynch et al., 2021; Jensen et al., 2016). In our study, autistic students were also characterized by higher intellectual abilities which might have activated the respondents’ positive beliefs toward autism. Such results support the idea that negative stereotypes and emphasis on socially unacceptable traits can contribute to increasing stigmatization; however, emphasis on positive traits might help in reducing social distancing (Link & Phelan, 2001; Lu et al., 2022).

It is important to note that higher levels of autism knowledge were associated with less desire for social distance in autism disclosure, non-disclosure, and autistic male conditions, but not in the autistic female condition. This adds to the existing literature, which has shown contradictory results regarding the relationship between autism knowledge and desired social distance (Gillespie-Lynch et al., 2015, 2021; Obeid et al., 2015; Someki et al., 2018; White et al., 2019), by suggesting that the type of knowledge, rather than its level, may contribute to social distancing. The Autism knowledge measure used in our study reflected a non-pathologizing view of autism, which is consistent with the literature stating that a more positive view of autism is associated with less stigma, while an emphasis on social inappropriateness might heighten social distance (Kitchin & Karlin, 2022; Lu et al., 2022; Matthews et al., 2015). Given that higher knowledge of autism was related to less social distancing from autistic males but not autistic females, it may suggest that the knowledge held by participants was still more representative of a typical male phenotype. Therefore, it shows the importance of including diverse autism presentations in autism awareness campaigns or students’ education programs.

Contrary to our expectations, prior contact with autistic individuals was not related to desired social distance from autistic students in any condition. This could be due to not considering the degree of closeness in contact during the analysis. Multiple studies show that having an autistic family member or friend reduces stigmatization (Gillespie-Lynch et al., 2015, 2017, 2022; Nevill & White, 2011). However, higher contact quality was related to less social distance when autism was disclosed. These findings contradict Link and Phelan’s (2001) statement that the diagnostic label enhances social distancing by activating negative stereotypes, but support the Intergroup contact theory, which suggests that the pleasantness of contact is a crucial factor determining the effect of contact on stigmatization (Allport, 1954). In our study, pleasant prior contact may have created positive associations with the autism label, decreasing social distance in disclosure conditions. However, in non-disclosure conditions, positive associations may not have been activated, and judgment may have been based on behavioral characteristics rather than prior experience with autism. Taking everything into consideration, we assume that closeness and pleasantness of prior contact may contribute to reducing social distancing. However, personal experience with autism may not be sufficient to reduce stigmatization when the diagnosis is unknown.

This study has several limitations. First, the generalizability of results is limited due to the high heterogeneity of autism expression in real life. The current findings can be best applied to autistic individuals of normal or higher intelligence with no visible behavioral issues. Including more diverse representations of autism is necessary to better understand the effect of different autism phenotypes on social distancing. Furthermore, this study focused only on explicit stigma. Some research shows that a decrease in explicit stigma is not necessarily related to a decrease in implicit stigma (Obeid et al., 2021). This limits the application of the results to situations where a person has time to consciously reflect on his behavior. In the case of automatic reactions, the results may not be easily generalized.

In addition, the answers to hypothetical scenarios may primarily reflect students’ self- perception and behavioral intentions, but not necessarily real-life behavior. While the gender variable was controlled in this study, the skewed female ratio may also limit the generalizability to the male population. This disproportion might have occurred as females are often more altruistic and willing to participate in online surveys (Brañas-Garza et al., 2018; Smith, 2008). Finally, unequal sample sizes with smaller samples in control groups might have affected the statistical power. Weak correlations of knowledge and contact of quality with the desire for social distance also suggest that caution is needed in interpreting the results.

In conclusion, this study contributes to the existing literature by examining different autism presentations. The findings showed that while students expressed less desire for social distance from autistic female students and autistic students with disclosed diagnoses compared to autistic males and those with non-disclosed diagnoses, students’ behavioral intentions toward hypothetical characters representing autistic females and autistic students with disclosed diagnoses did not differ significantly from those toward neurotypical students. Importantly, it does not imply that females with subtler autism expressions or those who disclose their diagnosis require less support. Although this study cannot reveal additional challenges that such students may face, it is crucial to acknowledge that non-disclosure and masking can be a result of autism-related stigma, hindering a sense of belonging (Anderson et al., 2017; Cassidy et al., 2020; Pearson & Rose, 2021; Perry et al., 2022). It highlights the need for further analysis of these groups to understand how to support them better. Furthermore, higher quality of prior contact with autism was related to less desire for social distance in autism-disclosed condition, and greater autism knowledge was linked to reduced social distance from autistic males and students with disclosed or non-disclosed diagnoses, but not autistic females. This emphasizes the need for higher education stigma reduction programs to facilitate positive interactions between groups and educate non-autistic students about different autism presentations, encompassing both challenges and strengths. Implementing these strategies and conducting further analysis of experiences across the broader autism spectrum can improve social inclusion programs, potentially enhancing the well-being, academic functioning, and satisfaction of university students (Ashbaugh et al., 2017; Davis et al., 2021; Gurbuz et al., 2019).

## References

[bibr1-13623613241230128] Allport G. W. (1954). The nature of prejudice. Addison-Wesley.

[bibr2-13623613241230128] Anderson A. H. Stephenson J. Carter M. (2017). A systematic literature review of the experiences and supports of students with autism spectrum disorder in post- secondary education. Research in Autism Spectrum Disorders, 39, 33–53. 10.1016/j.rasd.2017.04.002

[bibr3-13623613241230128] Ashbaugh K. Koegel R. Koegel L. (2017). Increasing Social integration for college students with autism spectrum disorder. Behavioral Development Bulletin, 22(1), 183–196. 10.1037/bdb0000057 28642808 PMC5476317

[bibr4-13623613241230128] Baron-Cohen S. Wheelwright S. Robinson J. Woodbury-Smith M. (2005). The Adult Asperger Assessment (AAA): A diagnostic method. Journal of Autism and Developmental Disorders, 35(6), 807–819. 10.1007/s10803-005-0026-5 16331530

[bibr5-13623613241230128] Beggiato A. Peyre H. Maruani A. Scheid I. Rastam M. Amsellem F. Gillberg C. I. Leboyer M. Bourgeron T. Gillberg C. Delorme R. (2017). Gender differences in autism spectrum disorders: Divergence among specific core symptoms. Autism Research: Official Journal of the International Society for Autism Research, 10(4), 680–689. 10.1002/aur.1715 27809408

[bibr6-13623613241230128] Bogardus E. S. (1933). A social distance scale. Sociology & Social Research, 17, 265–271.

[bibr7-13623613241230128] Botha M. Frost D. M. (2020). Extending the minority stress model to understand mental health problems experienced by the autistic population. Society and Mental Health, 10(1), 20–34. 10.1177/2156869318804297

[bibr8-13623613241230128] Brañas-Garza P. Capraro V. Rascon-Ramirez E. (2018). Gender differences in altruism on Mechanical Turk: Expectations and actual behaviour. Economics Letters, 170, 19–23. 10.1016/j.econlet.2018.05.022

[bibr9-13623613241230128] Brosnan M. Mills E. (2016). The effect of diagnostic labels on the affective responses of college students towards peers with ‘Asperger’s Syndrome’ and “Autism Spectrum Disorder.” Autism: The International Journal of Research and Practice, 20(4), 388–394. 10.1177/1362361315586721 26045542

[bibr10-13623613241230128] Cage E. Di Monaco J. Newell V. (2018). Experiences of autism acceptance and mental health in autistic adults. Journal of Autism and Developmental Disorders, 48(2), 473–484. 10.1007/s10803-017-3342-7 29071566 PMC5807490

[bibr11-13623613241230128] Cassidy S. A. Gould K. Townsend E. Pelton M. Robertson A. E. Rodgers J. (2020). Is Camouflaging autistic traits associated with suicidal thoughts and behaviours? Expanding the Interpersonal psychological theory of suicide in an undergraduate student sample. Journal of Autism and Developmental Disorders, 50(10), 3638–3648. 10.1007/s10803-019-04323-3 31820344 PMC7502035

[bibr12-13623613241230128] Cola M. L. Plate S. Yankowitz L. Petrulla V. Bateman L. Zampella C. J. de Marchena A. Pandey J. Schultz R. T. Parish-Morris J. (2020). Sex differences in the first impressions made by girls and boys with autism. Molecular Autism, 11(1), Article 49. 10.1186/s13229-020-00336-3 PMC7298946 32546266

[bibr13-13623613241230128] Cook J. Hull L. Crane L. Mandy W. (2021). Camouflaging in autism: A systematic review. Clinical Psychology Review, 89, 102080. 10.1016/j.cpr.2021.102080 34563942

[bibr14-13623613241230128] Davis M. T. Watts G. W. López E. J. (2021). A systematic review of firsthand experiences and supports for students with autism spectrum disorder in higher education. Research in Autism Spectrum Disorders, 84, 101769. 10.1016/j.rasd.2021.101769

[bibr15-13623613241230128] Evans S. C. Roberts M. C. Keeley J. W. Blossom J. B. Amaro C. M. Garcia A. M. Stough C. O. Canter K. S. Robles R. Reed G. M. (2015). Vignette methodologies for studying clinicians’ decision-making: Validity, utility, and application in ICD-11 field studies. International Journal of Clinical and Health Psychology, 15(2), 160–170. 10.1016/j.ijchp.2014.12.001 30487833 PMC6224682

[bibr16-13623613241230128] Gardiner E. Iarocci G. (2014). Students with autism spectrum disorder in the university context: Peer acceptance predicts intention to volunteer. Journal of Autism and Developmental Disorders, 44(5), 1008–1017. 10.1007/s10803-013-1950-4 24077739

[bibr17-13623613241230128] Geelhand P. Bernard P. Klein O. van Tiel B. Kissine M. (2019). The role of gender in the perception of autism symptom severity and future behavioral development. Molecular Autism, 10, Article 16. 10.1186/s13229-019-0266-4 PMC6439965 30976383

[bibr18-13623613241230128] Genevičiūtė-Janonienė G. (2015). Organizacinio įsipareigojimo kitimas ir reikšmė su darbuotoju ir organizacija susijusioms psichologinėms pasekmėms. Daktaro disertacija. Vytauto Didžiojo universitetas, Lietuva.

[bibr19-13623613241230128] Gillespie-Lynch K. Bisson J. B. Saade S. Obeid R. Kofner B. Harrison A. J. Daou N. Tricarico N. Delos Santos J. Pinkava W. Jordan A. (2022). If you want to develop an effective autism training, ask autistic students to help you. Autism: The International Journal of Research and Practice, 26(5), 1082–1094. 10.1177/13623613211041006 34472359

[bibr20-13623613241230128] Gillespie-Lynch K. Brooks P. J. Someki F. Obeid R. Shane-Simpson C. Kapp S. K. Daou N. Smith D. S. (2015). Changing college students’ conceptions of autism: An online training to increase knowledge and decrease stigma. Journal of Autism and Developmental Disorders, 45(8), 2553–2566. 10.1007/s10803-015-2422-9 25796194

[bibr21-13623613241230128] Gillespie-Lynch K. Daou N. Obeid R. Reardon S. Khan S. Goldknopf E. J. (2021). What contributes to stigma towards autistic university students and students with other diagnoses? Journal of Autism and Developmental Disorders, 51(2), 459–475. 10.1007/s10803-020-04556-7 32504342 PMC7273383

[bibr22-13623613241230128] Gillespie-Lynch K. Kapp S. K. Brooks P. J. Pickens J. Schwartzman B. (2017). Whose expertise is it? Evidence for autistic adults as critical autism experts. Frontiers in Psychology, 8, Article 438. 10.3389/fpsyg.2017.00438 PMC5368186 28400742

[bibr23-13623613241230128] Gurbuz E. Hanley M. Riby D. M. (2019). University students with autism: The social and academic experiences of university in the UK. Journal of Autism and Developmental Disorders, 49(2), 617–631. 10.1007/s10803-018-3741-4 30173311 PMC6373295

[bibr24-13623613241230128] Jensen C. M. Martens C. S. Nikolajsen N. D. Skytt Gregersen T. Heckmann Marx N. Goldberg Frederiksen M. Hansen M. S. (2016). What do the general population know, believe and feel about individuals with autism and schizophrenia: Results from a comparative survey in Denmark. Autism: The International Journal of Research and Practice, 20(4), 496–508. 10.1177/1362361315593068 26162627

[bibr25-13623613241230128] Jones E. E. (1984). Social stigma: The psychology of marked relationships. WH Freeman.

[bibr26-13623613241230128] Kitchin J. L. Karlin N. J. (2022). Awareness and stigma of autism spectrum disorders in undergraduate students. Psychological Reports, 125(4), 2069–2087. 10.1177/00332941211014144 33947290

[bibr27-13623613241230128] Link B. G. Phelan J. C. (2001). Conceptualizing stigma. Annual Review of Sociology, 27(1), 363–385.

[bibr28-13623613241230128] Link B. G. Yang L. H. Phelan J. C. Collins P. Y. (2004). Measuring mental illness stigma. Schizophrenia Bulletin, 30(3), 511–541. 10.1093/oxfordjournals.schbul.a007098 15631243

[bibr29-13623613241230128] Little L. M. Wallisch A. Salley B. Jamison R. (2017). Do early caregiver concerns differ for girls with autism spectrum disorders? Autism: The International Journal of Research and Practice, 21(6), 728–732. 10.1177/1362361316664188 27542396 PMC5532076

[bibr30-13623613241230128] Lu M. Wang R. Zou Y. Pang F. (2022). Chinese college students’ knowledge of autism spectrum disorder (ASD) and social distance from individuals with ASD: The mediating role of negative stereotypes. Journal of Autism and Developmental Disorders, 52(8), 3676–3685. 10.1007/s10803-021-05252-w 34453227

[bibr31-13623613241230128] Lynn M. R. (1986). Determination and quantification of content validity. Nursing Research, 35(6), 382–385. 3640358

[bibr32-13623613241230128] Matthews N. L. Ly A. R. Goldberg W. A. (2015). College students’ perceptions of peers with autism spectrum disorder. Journal of Autism and Developmental Disorders, 45(1), 90–99. 10.1007/s10803-014-2195-6 25070469

[bibr33-13623613241230128] McCrow J. Beattie E. Sullivan K. Fick D. M . (2013). Development and review of vignettes representing older people with cognitive impairment. Geriatric Nursing, 34(2), 128–137. 10.1016/j.gerinurse.2012.12.012 23347843

[bibr34-13623613241230128] Nevill R. E. White S. W. (2011). College students’ openness toward autism spectrum disorders: Improving peer acceptance. Journal of Autism and Developmental Disorders, 41(12), 1619–1628. 10.1007/s10803-011-1189-x 21318642 PMC13051561

[bibr35-13623613241230128] Obeid R. Bisson J. B. Cosenza A. Harrison A. J. James F. Saade S. Gillespie-Lynch K. (2021). Do implicit and explicit racial biases influence autism identification and stigma? An implicit association test study. Journal of Autism and Developmental Disorders, 51(1), 106–128. 10.1007/s10803-020-04507-2 32415531

[bibr36-13623613241230128] Obeid R. Daou N. DeNigris D. Shane-Simpson C. Brooks P. J. Gillespie-Lynch K. (2015). A cross-cultural comparison of knowledge and stigma associated with autism spectrum disorder among college students in Lebanon and the United States. Journal of Autism and Developmental Disorders, 45(11), 3520–3536. 10.1007/s10803-015-2499-1 26084712

[bibr37-13623613241230128] Paulhus D. L. (1991). Measurement and control of response bias. In Robinson J. P. Shaver P. R. Wrightsman L. S. (Eds.), Measures of personality and social psychological attitudes (pp. 17–59). Academic Press. 10.1016/B978-0-12-590241-0.50006-X

[bibr38-13623613241230128] Pearson A. Rose K. (2021). A conceptual analysis of autistic masking: Understanding the narrative of stigma and the illusion of choice. Autism in Adulthood, 3(1), 52–60. 10.1089/aut.2020.0043 36601266 PMC8992880

[bibr39-13623613241230128] Perry E. Mandy W. Hull L. Cage E. (2022). Understanding camouflaging as a response to autism-related stigma: A Social identity theory approach. Journal of Autism and Developmental Disorders, 52(2), 800–810. 10.1007/s10803-021-04987-w 33788076 PMC8813820

[bibr40-13623613241230128] Sasson N. J. Morrison K. E. (2019). First impressions of adults with autism improve with diagnostic disclosure and increased autism knowledge of peers. Autism: The International Journal of Research and Practice, 23(1), 50–59. 10.1177/1362361317729526 29039208

[bibr41-13623613241230128] Schuck R. K. Flores R. E. Fung L. K. (2019). Brief report: Sex/gender differences in symptomology and camouflaging in adults with autism spectrum disorder. Journal of Autism and Developmental Disorders, 49(6), 2597–2604. 10.1007/s10803-019-03998-y 30945091 PMC6753236

[bibr42-13623613241230128] Smith G . (2008). Does gender influence online survey participation? A record-linkage analysis of university faculty online survey response behavior. ERIC Document Reproduction Service No. ED 501717.

[bibr43-13623613241230128] Someki F. Torii M. Brooks P. J. Koeda T. Gillespie-Lynch K. (2018). Stigma associated with autism among college students in Japan and the United States: An online training study. Research in Developmental Disabilities, 76, 88–98. 10.1016/j.ridd.2018.02.016 29602160

[bibr44-13623613241230128] Stets J. E. Burke P. J. (2000). Identity theory and social identity theory. Social Psychology Quarterly, 63(3), 224–237. 10.2307/2695870

[bibr45-13623613241230128] Stockwell K. M. Bottini S. Jaswal V. K. Gillis J. M. (2021). Brief report: Social behavior and special interests in the stigmatization of autistic college students. Journal of Autism and Developmental Disorders, 51(9), 3356–3364. 10.1007/s10803-020-04769-w 33146877

[bibr46-13623613241230128] Stone W. L. (1987). Cross-disciplinary perspectives on autism. Journal of Pediatric Psychology, 12(4), 615–630. 10.1093/jpepsy/12.4.615 3323447

[bibr47-13623613241230128] Tillmann J. Ashwood K. Absoud M. Bölte S. Bonnet-Brilhault F. Buitelaar J. K. Calderoni S. Calvo R. Canal-Bedia R. Canitano R. De Bildt A. Gomot M. Hoekstra P. J. Kaale A. McConachie H. Murphy D. G. Narzisi A. Oosterling I. Pejovic-Milovancevic M. . . .Charman T. (2018). Evaluating sex and age differences in ADI-R and ADOS scores in a large European multi-site sample of individuals with autism spectrum disorder. Journal of Autism and Developmental Disorders, 48(7), 2490–2505. 10.1007/s10803-018-3510-4 29468576 PMC5996001

[bibr48-13623613241230128] Underhill J. C. Ledford V. Adams H. (2019). Autism stigma in communication classrooms: Exploring peer attitudes and motivations toward interacting with Atypical students. Communication Education, 68(2), 175–192. 10.1080/03634523.2019.1569247

[bibr49-13623613241230128] Waltz C. F. Strickland O. L. Lenz E. R. (2005). Measurement in nursing and health research (3rd ed.). Springer.

[bibr50-13623613241230128] Weiner B. (1993). On sin versus sickness. A theory of perceived responsibility and social motivation. The American Psychologist, 48(9), 957–965. 10.1037//0003-066x.48.9.957 8214914

[bibr51-13623613241230128] White D. Hillier A. Frye A. Makrez E. (2019). College students’ knowledge and attitudes towards students on the autism spectrum. Journal of Autism and Developmental Disorders, 49(7), 2699–2705. 10.1007/s10803-016-2818-1 27230760

[bibr52-13623613241230128] Whitlock A. Fulton K. Lai M. C. Pellicano E. Mandy W. (2020). Recognition of girls on the autism spectrum by primary school educators: An experimental study. Autism Research: Official Journal of the International Society for Autism Research, 13(8), 1358–1372. 10.1002/aur.2316 32488964

[bibr53-13623613241230128] Wood C. Freeth M. (2016). Students’ stereotypes of autism. Journal of Educational Issues, 2(2), 131–140. 10.5296/jei.v2i2.997

